# Approach to the patient: precision medicine–guided evaluation and treatment of acromegaly

**DOI:** 10.1210/clinem/dgag241

**Published:** 2026-06-18

**Authors:** Manel Puig-Domingo, Montserrat Marques-Pamies, Miguel Sampedro, Joan Gil, Marta Araujo-Castro, Rebeca Martínez-Hernández, Betina Biagetti, Mireia Jordà, Elena Valassi, Mónica Marazuela

**Affiliations:** Endocrinology and Nutrition Service, Hospital Universitari Germans Trias i Pujol, Badalona 08916, Spain; Endocrine Tumors Research Group, Germans Trias i Pujol Research Institute, Badalona 08916, Spain; Department of Medicine, Universitat Autònoma de Barcelona, Campus de can Ruti, Badalona 08916, Spain; Endocrine Tumors Research Group, Germans Trias i Pujol Research Institute, Badalona 08916, Spain; Endocrinology and Nutrition Service, Hospital de Granollers, Granollers 08402, Spain; Endocrinology and Nutrition Service, Hospital Universitario la Princesa, Madrid 28006, Spain; Endocrine Tumors Research Group, Germans Trias i Pujol Research Institute, Badalona 08916, Spain; CIBERER Group 747, Instituto de Salud Carlos III, Madrid 28029, Spain; Endocrinology and Nutrition Service, Hospital Universitario Ramón y Cajal, Madrid 28034, Spain; Endocrinology and Nutrition Service, Hospital Universitario la Princesa, Madrid 28006, Spain; Endocrinology and Nutrition Service, Hospital Universitari Vall D’Hebron, Barcelona 08035, Spain; Endocrine Research Group, Vall D’Hebron Research Institute, Barcelona 08035, Spain; Endocrine Tumors Research Group, Germans Trias i Pujol Research Institute, Badalona 08916, Spain; Endocrinology and Nutrition Service, Hospital Universitari Germans Trias i Pujol, Badalona 08916, Spain; Endocrine Tumors Research Group, Germans Trias i Pujol Research Institute, Badalona 08916, Spain; CIBERER Group 747, Instituto de Salud Carlos III, Madrid 28029, Spain; Department of Medicine, Universitat Internacional de Catalunya, Barcelona 08017, Spain; Endocrinology and Nutrition Service, Hospital Universitario la Princesa, Madrid 28006, Spain; Instituto de Investigación Sanitaria la Princesa, Madrid 28006, Spain; Department of Medicine, Universidad Autónoma de Madrid, Madrid 28029, Spain

**Keywords:** precision medicine, acromegaly, biomarkers, imaging, functional tests, molecular profiling

## Abstract

Acromegaly is a heterogeneous disease in which delayed biochemical control remains common despite the availability of multiple therapeutic options. Traditional stepwise medical treatment algorithms often rely on the use of first-generation somatostatin receptor ligands (fgSRLs) as first-line drugs with empirical escalation and trial-and-error approaches, prolonging patients' exposure to hormonal excess.

Cluster analyses indicate that overall, 3 main classes of patients with acromegaly representing distinct biological phenotypes can be recognized: (1) young patients with invasive macroadenomas and frequent resistance to fgSRLs; (2) older patients with noninvasive tumors and SRL responsiveness in the majority of them; and (3) patients with intermediate features often requiring combination medical therapy in whom prediction of response is more challenging.

From a practical perspective, biomarker-guided treatment selection based on T2-weighted magnetic resonance imaging signal intensity, the short acute octreotide test, and tumor immunohistochemistry, following the strategy validated in the ACROFAST study, is applicable in clinical practice. It allows prompt biochemical control and tumor volume reduction, a rationale leading to superior effectiveness performance than the classic sequencing therapy involving the universal utilization of fgSRLs as first option.

In the era of integrative and participative medicine, such a protocol enables the early identification of most patients unlikely to respond to fgSRLs, thus supporting timely initiation of pegvisomant, pasireotide, or combination therapy, and the achievement of hormonal control in nearly 80% of patients.

*Conclusion* Precision medicine has become a practical reality in acromegaly. A biomarker-guided strategy improves therapeutic efficiency and should be incorporated into contemporary clinical practice.

## Introduction and biological rationale for precision medicine in acromegaly

Acromegaly is a rare and invalidating endocrine disorder caused by chronic exposure to excessive growth hormone (GH) and insulin-like growth factor 1 (IGF-1), most commonly resulting from a GH-secreting pituitary neuroendocrine tumor (PitNET) (1). The disease is insidious in onset, frequently diagnosed after a prolonged latency of several years, during which progressive somatic disfigurement, metabolic derangements, and multisystemic complications develop. Epidemiological studies have demonstrated that uncontrolled acromegaly is associated with increased morbidity, impaired quality of life (QoL), and premature mortality, largely driven by cardiovascular disease, respiratory complications, diabetes mellitus, and malignancy, whereas biochemical normalization of IGF-1 and GH restores survival to that of the general population (2-4).

Over recent decades, major therapeutic advances have transformed acromegaly from a largely uncontrolled disease into one for which long-term biochemical remission is achievable in most patients (5). Improvements in endoscopic transsphenoidal surgery, together with the availability of effective medical therapies, including first-generation somatostatin receptor ligands (fgSRLs), dopamine agonists, the GH receptor antagonist pegvisomant, and the multireceptor somatostatin ligand pasireotide have markedly increased overall control rates. In addition, repeat transsphenoidal surgery in selected cases and improvements in stereotactic radiosurgery techniques have further expanded multimodal treatment options, particularly for local tumor control, while biochemical benefit is often delayed. Nonetheless, real-world registries and cohort studies have indicated that a substantial proportion of patients experience difficulties in achieving rapid biochemical control, often requiring multiple therapeutic modifications over even several years until hormonal control (6, 7).

Failure to achieve a rapid biochemical control prolongs exposure to elevated GH/IGF-1, thus perpetuating cardiovascular remodeling, insulin resistance, arthropathy, and reduced QoL, and is associated with increased health-care utilization (8). A major contributor to this delay is the traditional stepwise, trial-and-error treatment paradigm, in which all patients are treated with fgSRLs as a first-line medical option irrespective of the patient's characteristics and tumor biology, followed by sequential dose escalation, switching, or add-on therapies based on biochemical response assessed months later.

In parallel, a growing body of evidence has confirmed that acromegaly is a biologically heterogeneous disease. Somatotroph PitNETs vary in terms of cellular differentiation state, granulation pattern, somatostatin receptor expression profiles, intracellular signaling pathways, invasiveness, and responsiveness to pharmacological agents (9, 10). Importantly, several of these features can be assessed even before surgery, at the time of diagnosis through noninvasive imaging and functional testing, specifically the standardized short acute octreotide test (sAOT), which assesses acute GH suppression after subcutaneous octreotide as an in vivo indicator of somatostatin receptor responsiveness. This recognition has paved the way for precision medicine approaches aimed at individualizing treatment selection and particularly medical therapy based on patient- and tumor-specific characteristics (5, 11, 12).

Over the last decade, our group has contributed to obtaining key evidence supporting the clinical applicability of such an approach. These contributions include the demonstration that T2-weighted magnetic resonance imaging (MRI) signal intensity identifies response to fgSRL and pasireotide therapy (13-15), the validation of the sAOT as a functional predictor of long-term response to fgSRLs (16), and the integration of tumor biomarkers such as E-cadherin and somatostatin receptor subtype 2 (SSTR2) as pathological correlates of medical treatment sensitivity (17).

Most important, these concepts have been prospectively translated into clinical practice in the ACROFAST study (Acromegaly Clinical Research on Outcomes with Functional and Stratified Therapy), a multicenter prospective trial comparing a biomarker-driven personalized treatment algorithm with standard guideline-based care. In this study, treatment selection was guided by predictive biomarkers of response to fgSRL, including the sAOT, T2-weighted MRI tumor signal, and E-cadherin expression. This personalized strategy demonstrated significantly higher rates of hormonal control and a shorter time to disease control compared with standard care based on uniform first-line fgSRL therapy (18, 19).

The present paper builds on that framework and illustrates how precision medicine principles can be operationalized in routine clinical care.

## T2-weighted magnetic resonance imaging signal as a noninvasive biomarker

### Biological basis

T2-weighted MRI signal intensity reflects tissue water content, cellular density, and intracellular protein composition. In somatotroph PitNETs, T2 signal has been shown to strongly correlate with granulation pattern: Densely granulated tumors tend to be T2-hypointense, whereas sparsely granulated tumors are more often T2-isointense or hyperintense. This relationship provides a radiological window into tumor biology that can be exploited for treatment stratification (20).

### Clinical validation

The predictive value of T2-weighted MRI signal was first demonstrated by our group in a study showing that patients with T2-hypointense adenomas had significantly higher rates of biochemical response to fgSRLs after surgical failure than those with T2-hyperintense tumors (13, 21). This observation was subsequently validated in independent cohorts and multicenter studies, which confirmed that T2 hypointensity predicts not only biochemical control but also greater tumor shrinkage under fgSRL therapy (22).

Importantly, T2 signal intensity is obviously available at diagnosis, before any therapeutic intervention, making it an ideal biomarker for tumor classification and useful for early therapeutic decision-making. Its predictive value appears robust across different MRI platforms and acquisition protocols, enhancing its generalizability.

## Biological heterogeneity of somatotroph pituitary neuroendocrine tumors

### Granulation pattern and tumor differentiation

**Figure 1:**
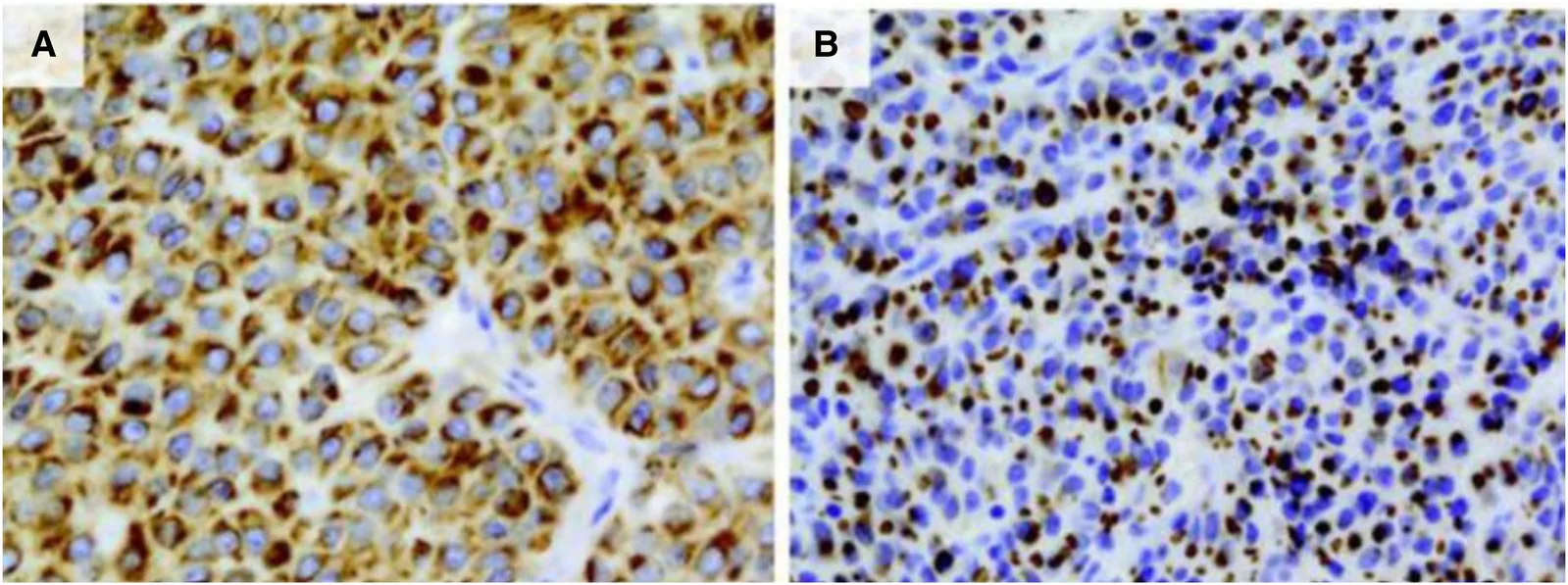
A, Densely granulated somatotroph pituitary neuroendocrine tumor (PitNET) with strong, diffuse cytoplasmic growth hormone (GH) immunoreactivity and a perinuclear/diffuse cytoplasmic CAM5.2 staining pattern. B, Sparsely granulated somatotroph PitNET showing weak and heterogeneous GH expression with characteristic dot-like paranuclear CAM5.2–positive fibrous bodies. Intermediate tumors typically show mixed keratin staining, with interspersed dot-like and perinuclear patterns.

One of the most fundamental sources of heterogeneity in acromegaly lies in the histological differentiation of somatotroph PitNETs. These tumors are classically divided into densely granulated and sparsely granulated adenomas, based on cytokeratin staining patterns and ultrastructural features (Fig. 1) (23). Densely granulated tumors typically occur in older patients, exhibit slower growth, higher expression of SSTR2 and E-cadherin, and respond more favorably to fgSRL therapy. In contrast, sparsely granulated tumors are more common in younger patients, often present with larger and more invasive adenomas, show lower SSTR2 expression and E-cadherin, and are quite resistant to fgSRLs (24, 25).

While granulation pattern is a powerful predictor of treatment response, it is typically available only after surgical resection. This limitation has driven interest in identifying noninvasive surrogates of tumor differentiation that can be assessed at diagnosis.

### Linking imaging, receptor biology, and treatment response

Mechanistically, the association between T2 signal and fgSRL responsiveness is explained by receptor expression and intracellular signaling. Densely granulated, T2-hypointense tumors typically express high levels of SSTR2, the primary mediator of the antisecretory and antiproliferative effects of fgSRLs. Conversely, sparsely granulated, T2-isointense or hyperintense tumors often show reduced SSTR2 expression and altered downstream signaling, limiting their responsiveness to these agents (12). These observations underscore why a “one-size-fits-all” approach to fgSRL therapy is suboptimal and imaging biomarkers should be integrated into initial treatment planning (5).

## Functional prediction of medical response and precision-guided management of invasive disease. The short acute octreotide test: physiological rationale and clinical validation

### Historical background and initial skepticism

The concept of using an acute somatostatin analogue challenge to predict long-term responsiveness in acromegaly dates back to the early era of medical therapy, when subcutaneous octreotide was first introduced. Early studies explored whether short-term GH suppression following octreotide administration could anticipate chronic treatment efficacy, and basically, to assess how many injections of subcutaneous octreotide a given patient would require. However, results were heterogeneous, protocols were sometimes inconsistent, and correlations with long-term outcomes were unsatisfactory. In particular, acute tests varied in dose, timing, and end points; GH assays lacked standardization; and most studies did not account for tumor heterogeneity, and did not integrate imaging and pathological correlates. Importantly, acute testing was often interpreted in isolation, without reference to tumor anatomy, MRI characteristics, or molecular phenotype (26). As a consequence, acute testing gradually fell out of favor and was largely abandoned in international guidelines (27, 28).

### Renewed interest and modern reinterpretation

Interest in acute functional testing has been renewed in recent years as the understanding of somatotroph tumor biology has advanced. It is now recognized that responsiveness to fgSRLs is determined not only by receptor expression but also by receptor coupling, intracellular signaling integrity, and cellular differentiation state. In this context, a standardized short version of 2 hours instead of 6 hours’ duration—as originally described—currently known as the sAOT, can be viewed as a real-time pharmacodynamic probe of tumor sensitivity to somatostatin receptor activation. In the last decade two studies have proven its consistency and high predictive value, with areas under the receiver operating characteristic curve largely greater than 0.8 (29, 30).

Our group has recently revisited the sAOT performance within a modern precision medicine framework, using a standardized protocol (100-µg subcutaneous octreotide with GH measurement at baseline and 2 hours) and correlating results with long-term biochemical response, MRI characteristics, and tumor immunohistochemistry with the ACROFAST cohort (16). In that multicenter study, GH_2h_ less than 1.4 ng/mL showed the highest ability to identify responders (accuracy of 81%, sensitivity of 73.3%, specificity of 94.1%). GH_2h_ greater than 4.3 ng/mL was the best cutoff for nonresponse prediction (accuracy of 74%, sensitivity of 35.3%, specificity of 96.7%); thus, poor GH suppression during sAOT was associated with reduced likelihood of achieving IGF-1 normalization under fgSRLs, whereas robust suppression predicted favorable response (area under the receiver operating characteristic curve = 0.83). However, these cutoffs should be interpreted according to the baseline GH values. Thus, in patients with very high baseline GH concentrations, substantial relative suppression may still indicate biological responsiveness even if the 2-hour GH value does not fall below fixed thresholds predicting full response to fgSRLs; these patient cases are considered as partial responders; they benefit from fgSRLS treatment but require combined treatment with either pegvisomant and/or cabergoline for achieving normalization of IGF1, or they may eventually be cases with complete response to pasireotide. Therefore, baseline GH values and the magnitude of Δ GH reduction should be important elements when interpreting the sAOT, particularly in severe biochemical phenotypes.

The postoperative interpretation of sAOT results may not be identical to the preoperative setting, as debulking reduces secretory tumor mass and may modify the suppressibility of the octreotide challenge to the current residual tumor. Therefore, due to the simplicity of the sAOT, repeating the test after surgery is recommended when substantial mass debulking has been possible.

### Complementarity with imaging biomarkers

Importantly, the sAOT does not replace imaging biomarkers such as T2-weighted MRI signal intensity but rather complements them. While T2 signal reflects intrinsic tumor properties linked to granulation pattern and receptor expression, the sAOT captures functional responsiveness of the tumor to somatostatin receptor activation. This distinction is particularly valuable in intermediate or discordant phenotypes, in which imaging and clinical features alone may not provide a clear prediction.

In the ACROFAST study, integration of sAOT results with MRI characteristics allowed early stratification of patients into treatment pathways that minimized exposure to ineffective therapy and accelerated biochemical control.

## Patient 1

### A young man with aggressive, invasive disease, and predicted resistance to first-generation somatostatin receptor ligands

#### Clinical presentation

A 25-year-old man was referred to our pituitary center for evaluation of progressive somatic changes and systemic symptoms. Over the preceding 4 years, he had noted enlargement of his hands and feet, necessitating repeated changes in shoe and ring size, progressive facial coarsening, mandibular prognathism, and spacing of the teeth. He also reported abnormal hyperhidrosis, frequent headaches with retro-orbital pressure, arthralgia involving the knees and wrists, and recent onset of hypertension requiring pharmacological treatment.

Additional symptoms included fatigue, reduced exercise tolerance, and intermittent paresthesia of the hands consistent with carpal tunnel syndrome. His partner reported loud snoring and witnessed episodes of apnea, raising suspicion of obstructive sleep apnea. There was no known family history of pituitary disease or endocrine neoplasia.

#### Laboratory evaluation

Biochemical testing revealed a markedly elevated age-adjusted IGF-1 level (2.3 × upper limit of normal [ULN]). Random GH concentrations were persistently elevated (>9 ng/mL), and GH failed to suppress during an oral glucose tolerance test (2-hour nadir of GH = 5.2 ng/mL). Prolactin (PRL) levels were mildly elevated (35 ng/mL; normal values, 5-18 ng/mL), consistent with stalk compression effect. Evaluation of other pituitary axes showed borderline secondary hypogonadism, with low to normal testosterone and inappropriately normal gonadotropins, while thyroid and adrenal axes were preserved.

Metabolic evaluation demonstrated impaired fasting glucose, elevated glycated hemoglobin A_1c_ (HbA_1c_) in the prediabetes range (6.1%), dyslipidemia with elevated triglycerides, and evidence of left ventricular hypertrophy on echocardiography. Polysomnography confirmed moderate obstructive sleep apnea.

#### Imaging findings

**Figure 2:**
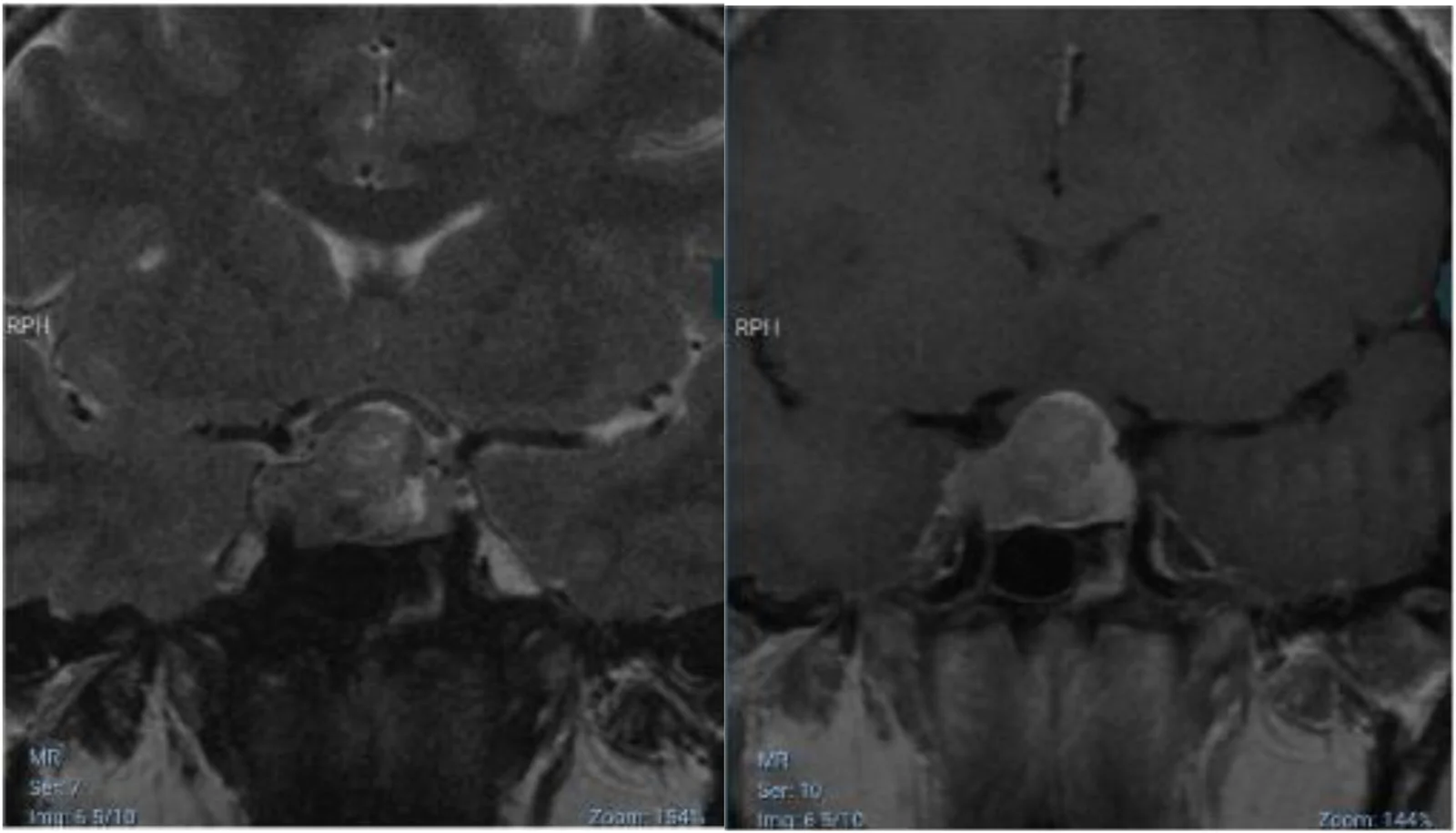
Magnetic resonance imaging of patient 1: pituitary adenoma showing a heterogeneous hyperintense signal in the T2-weighted sequence with invasion to the right cavernous sinus.

Pituitary MRI revealed a heterogeneous 3-cm macroadenoma with significant cavernous sinus right side invasion (Knosp grade 3-4) (Fig. 2). There was no optic chiasm compression. On T2-weighted sequences, the tumor displayed a markedly hyperintense signal, a feature associated with sparse granulation and reduced responsiveness to fgSRLs.

#### Functional testing: short acute octreotide test

A standardized sAOT was performed. GH decreased from 18.4 ng/mL at baseline to 5.6 ng/mL at 2 hours, remaining well above 4.3 ng/mL, a threshold associated with poor long-term response to fgSRLs according to our experience (16). This finding showed a marked but insufficient acute suppression and a very low probability of achieving adequate biochemical control with fgSRL monotherapy.

#### Surgical decision-making and rationale for debulking

Given the young age of the patient, considerable symptom burden, and aggressive tumor anatomy, transsphenoidal surgery was recommended. However, complete resection was considered unlikely due to extensive cavernous sinus invasion. The surgical goal was therefore maximal safe debulking, aimed at reducing tumor mass, alleviating local symptoms, and facilitating postoperative medical control. After surgery, it was estimated that about 75% of the tumor mass was resected and a 0.8-cm maximal diameter tumor rest adhering to the intracavernous right side carotid artery was left.

The rationale for debulking invasive GH-producing PitNETs is multifaceted. Reducing tumor burden can improve hormonal control by decreasing the total secretory mass, may enhance responsiveness to adjuvant medical therapy, and can mitigate long-term risks associated with tumor progression. Several studies have demonstrated that surgical debulking improves subsequent medical outcomes in some patients (31), even when biochemical cure is not achieved (32, 33). Thus, high levels of RAR-related orphan receptor C (RORC)—a nuclear transcription factor closely related to E-cadherin expression—and low levels of Ki-67 identify improved fgSRL response after surgical debulking in large somatotropic adenomas (31), which was not the case for this patient.

#### Postoperative pathology and tumor phenotype

**Figure 3:**
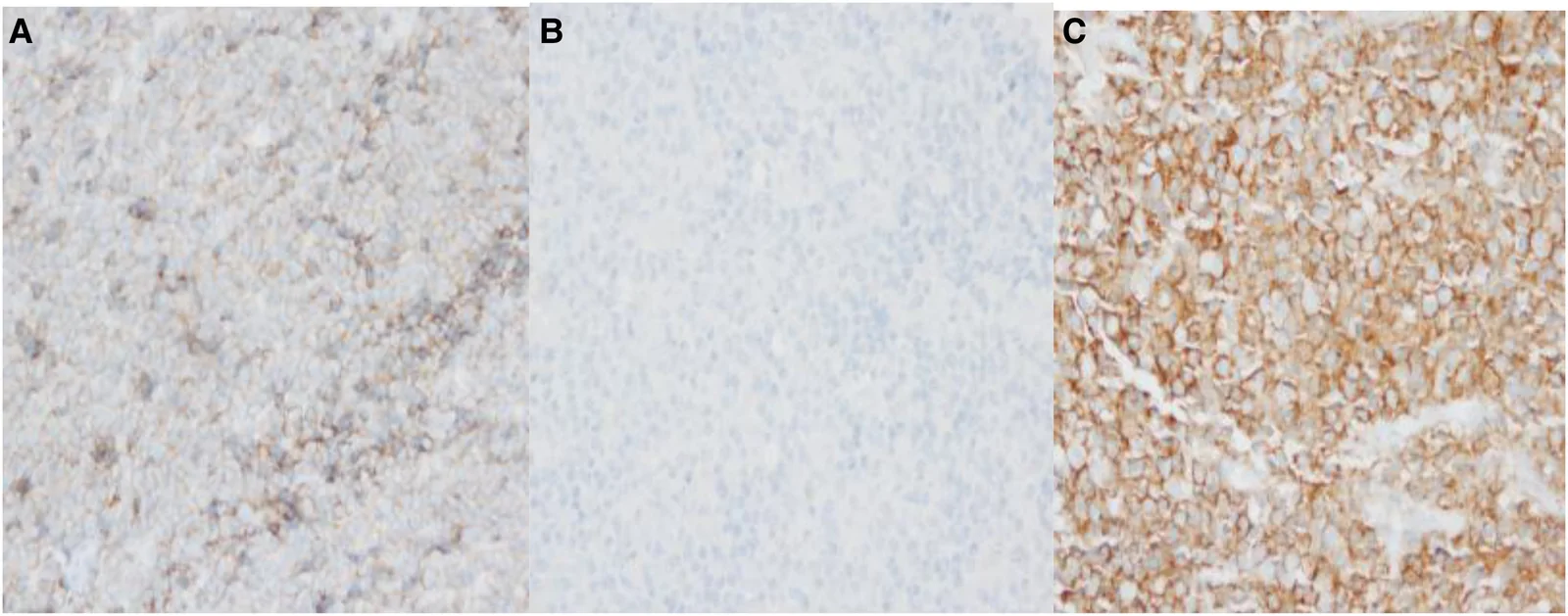
E-cadherin. immunohistochemistry (×200). A, Tumor from patient 1 showing low expression; B, tumor with negative staining; C, tumor with strong positivity.

Histopathological examination confirmed a sparsely granulated somatotroph PitNET staining diffusely for GH and not for PRL. Immunohistochemistry also demonstrated low E-cadherin (Fig. 3), consistent with a more invasive phenotype, and low SSTR2 expression, both supporting the predicted poor response to fgSRLs (10). Ki-67 index was modestly elevated (4%), reflecting increased proliferative activity. Moreover, *AIP* mutations and AIP immunohistochemical expression were also studied and a nonsense *AIP* germ-line mutation was found together with very low AIP immunohistochemical staining. All first-degree family members were thereafter evaluated to determine carrier status without evidence of mutation in all of them.

#### Precision-guided postoperative treatment selection

In traditional treatment algorithms, postoperative management of this patient would typically have involved initiation of fgSRLs, with dose escalation over several months to assess response. However, based on concordant predictive markers—T2 hyperintensity, a new sAOT performed after surgery showing a nonsuppression value of 5.1 ng/mL, and an unfavorable tumor immunophenotype with low E-cadherin expression and an *AIP* mutation—this patient was classified as very unlikely to benefit from fgSRL monotherapy.

Following the ACROFAST protocol and study results, in which an sAOT GH nadir greater than 4.3 ng/mL and T2 hyperintense signal identify a very poor response to fgSRLs, pegvisomant was initiated early after surgery as a first-line postoperative medical therapy and was titrated gradually, leading to normalization of IGF-1 within 3 months at a dose of 0.2 mg/kg/day, which was given with a frequency regimen of 1 injection every 48 hours. Liver function tests remained normal, and serial MRI showed stable residual tumor without evidence of progression during the following follow-up year. After 12 months of treatment, the patient requested an alternative medical treatment that could alleviate the number of injections per week. Pasireotide therapy was discussed, taking into account that it has shown a 60% positive response rate in fgSRL-resistant patients, even in the presence of *AIP* mutations (34). Moreover, new immunohistochemical assessment of the tumor showed a mid-intensity positive expression of SSTR5, supporting the indication of pasireotide. After progressive withdrawal of pegvisomant, at 6 months of pasireotide with a dose of 20 mg/month, IGF-1 was maintained within the normal range and the tumor showed further reduction to a detectable 0.4 cm as the largest diameter. However, glycemic control deteriorated with the patient’s HbA_1c_ increasing to 7.9%, and diabetes treatment initially required a dipeptidyl peptidase 4 (DPP-4) inhibitor compound, followed by a weekly glucagon-like peptide-1 receptor agonist plus metformin to go back to an HbA_1c_ of 6.5%. Radiotherapy was proposed 12 months later after initiation of pasireotide after discussing this option with the patient to obtain definitive remission. Stereotaxic radiotherapy was considered safe enough to preserve the normal pituitary parenchyma as the tumor remnant measured only 0.4 cm. At this time there was not sufficient clinical experience with the long-term tumoricidal effect of pasireotide, which is now more well known. At present, a patient case such as this young man would have also been treated with pasireotide to look for continued reduction of tumor remnant and eventual tumor disappearance and hormonal remission.

#### Clinical impact

Rapid biochemical control was accompanied by marked clinical improvement, including reduction in headaches, improved blood pressure control, partial regression of soft tissue swelling, and improved glycemic parameters before initiation of pasireotide. This case illustrates how early identification of fgSRL-resistant phenotypes allows clinicians to bypass ineffective therapies and minimize cumulative exposure to excess GH and IGF-1, even in the worst biological scenarios, including *AIP* mutation. Germline *AIP* mutations identify a subgroup of somatotroph tumors characterized by earlier onset, larger and more invasive adenomas, and lower responsiveness to first-generation SRLs. This association is clinically important because it may partly explain primary or marked reduced response or true resistance to fgSRLs in young patients with aggressive disease due to an altered receptor downstream G-protein pathway. Nevertheless, *AIP* mutation testing should not be triggered by fgSRL resistance alone. Current recommendations suggest considering sequential genetic testing for AIP (and then multiple endocrine neoplasia type 1, unless syndromic multiple endocrine neoplasia type 1 features are present) mainly in patients with a family history of pituitary tumors, disease onset at 18 years or younger, pituitary gigantism, or GH-secreting macroadenoma diagnosed at 30 years or younger; selected patients with multiple pituitary tumors may also merit testing.

## Patient 2

### Precision medicine in favorable cases: delayed surgery and preoperative therapy in an older woman with favorable biomarkers profile and sustained response to first-generation somatostatin receptor ligand therapy

#### Clinical presentation

A 76-year-old woman was referred for endocrine evaluation following incidental recognition of acromegalic features by her primary care physician during assessment of long-standing type 2 diabetes mellitus and recently diagnosed obstructive sleep apnea. After clinical suspicion by her physician, on careful questioning, the patient reported progressive enlargement of her hands and feet over approximately 8 to 10 years, necessitating larger shoe sizes, as well as mild facial coarsening—as assessed by pictures obtained from her family album—increased interdental spacing, and chronic fatigue. She denied substantial headaches or visual disturbances. Musculoskeletal complaints were limited to mild arthralgia of the knees and hips; the patient attributed all the clinical picture to aging.

Her past medical history was notable for type 2 diabetes mellitus treated with metformin and a DPP-4 inhibitor, arterial hypertension controlled with a single antihypertensive agent, and dyslipidemia. There was no family history of pituitary or endocrine disease.

#### Laboratory evaluation

Biochemical testing revealed an age-adjusted IGF-1 concentration 1.6 times the ULN. Random GH levels were elevated but lower than typically observed in aggressive disease (4-7 ng/mL). GH failed to suppress adequately during an oral glucose tolerance test (GH 2 hours = 3.8 ng/mL), confirming the diagnosis of acromegaly. PRL levels were normal. The other pituitary axes were intact. Metabolic evaluation showed a moderately elevated HbA_1c_ (7.4%), mild hypertriglyceridemia, and normal liver enzymes. Echocardiography demonstrated mild concentric left ventricular hypertrophy without systolic dysfunction.

#### Imaging findings

**Figure 4:**
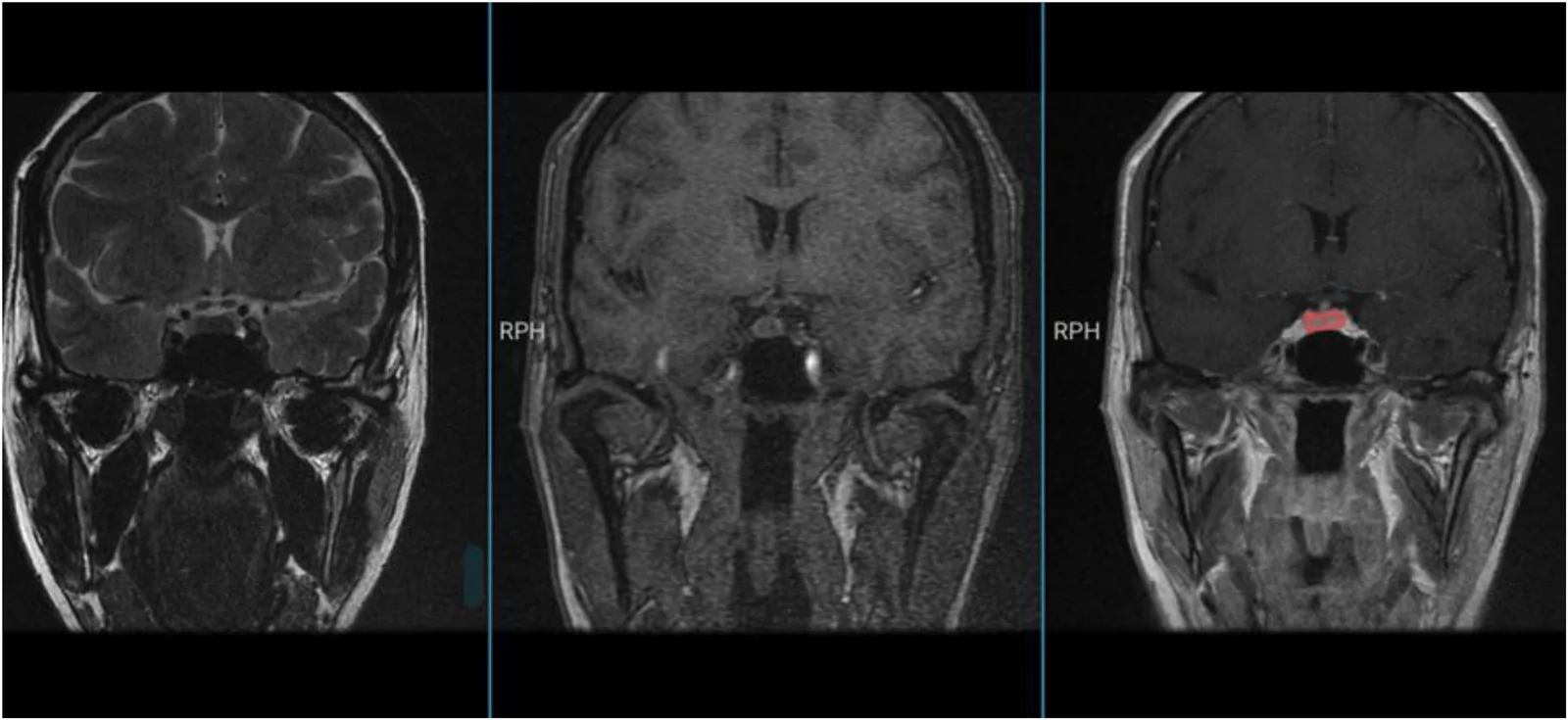
Magnetic resonance imaging of patient 2: flat pituitary adenoma with 1.1-cm largest diameter showing a hypointense signal obtained in the T2-weighted sequence.

Pituitary MRI showed a 1.2-cm maximal diameter flat centered adenoma confined to the sella, without cavernous sinus invasion or suprasellar extension (Fig. 4). Importantly, the tumor exhibited a markedly hypointense signal on T2-weighted sequences, a feature strongly associated with densely granulated somatotroph tumors and favorable response to fgSRLs.

#### Functional testing: short acute octreotide test

A standardized sAOT was performed. Baseline GH was 6.2 ng/mL and decreased to 0.8 ng/mL at 2 hours, demonstrating robust acute suppression by subcutaneous octreotide. This degree of suppression has been consistently associated with high a likelihood of biochemical control under chronic fgSRL therapy.

#### Surgical considerations and patient preference

Transsphenoidal surgery was recommended as first-line and curative therapy, given the noninvasive tumor anatomy and reasonable probability of surgical remission. However, after extensive discussion, the patient expressed substantial apprehension regarding surgery and requested time to consider her options. She was particularly concerned about perioperative risks and the potential effect on QoL.

In this context, medical therapy was considered both as a temporizing measure and as a potential long-term strategy, provided that hormonal control could be achieved.

#### Precision-guided medical therapy

Based on the concordantly favorable biomarkers—T2 hypointensity and robust sAOT suppression—the patient was classified as highly likely to respond to fgSRL therapy. Octreotide LAR 20 mg every 4 weeks was initiated. By the fourth month of therapy, her IGF-1 had normalized and GH levels decreased to less than 1 ng/mL without dose escalation. The patient reported gradual improvement in fatigue, reduction in soft tissue swelling, and better glycemic control, allowing simplification of her antidiabetic regimen with even discontinuation of DPP-4 inhibitor therapy.

#### Follow-up and clinical implications

Repeated MRI after 12 months showed a tumor size decrease of about 30%. Given sustained biochemical control and tumor shrinkage, the patient elected to continue medical therapy rather than proceed with surgery. This case illustrates how precision biomarkers can justify confident use of fgSRL monotherapy, even when surgery is deferred, and underscores the importance of individualized decision-making rather than rigid adherence to algorithmic sequencing.

These observations are consistent with outcomes reported in the ACROFAST study, in which patients with favorable biomarker profiles achieved rapid and sustained control on fgSRL therapy.

## Patient 3

### Intermediate phenotype with high tumor burden: rationale for preoperative combination therapy

#### Clinical presentation

A 48-year-old man with family antecedents of type 2 diabetes presented with an apparently 2-year history of progressively worsening headaches, marked hyperhidrosis, arthralgia affecting the shoulders, knees, and hands, profound fatigue, and decreased libido. He reported a substantial increase in shoe and glove size over a relatively short period, accompanied by worsening snoring, daytime somnolence, and impaired concentration. He was also diagnosed with type 2 diabetes that required recent initiation of basal insulin therapy, and dapagliflozin added later on (35). He also presented with mild hypertension with blood pressure values higher than 150/95 mm Hg on repeated occasions that required pharmacologic therapy with enalapril and nicardipine.

On physical examination, he exhibited prominent acromegalic features, including frontal bossing, prognathism, macroglossia, and thickened skin. Neurological examination revealed no visual field deficits, although the patient reported intermittent blurred vision during headaches.

#### Laboratory evaluation

IGF-1 concentration was markedly elevated at approximately 2.0 times the ULN. Random GH values ranged between 10 and 14 ng/mL, and GH failed to suppress during oral glucose tolerance testing. PRL levels were abnormal, with values ranging from 125 to 170 ng/mL. Evaluation of pituitary axes revealed secondary hypogonadism with low testosterone values, while thyroid and adrenal function were preserved.

Metabolic assessment demonstrated poorly controlled diabetes mellitus (HbA_1c_ 8.6%), hypertriglyceridemia, and mild elevation of liver transaminases consistent with metabolic-associated fatty liver disease. Echocardiography showed concentric left ventricular hypertrophy with early diastolic dysfunction.

#### Imaging findings

**Figure 5:**
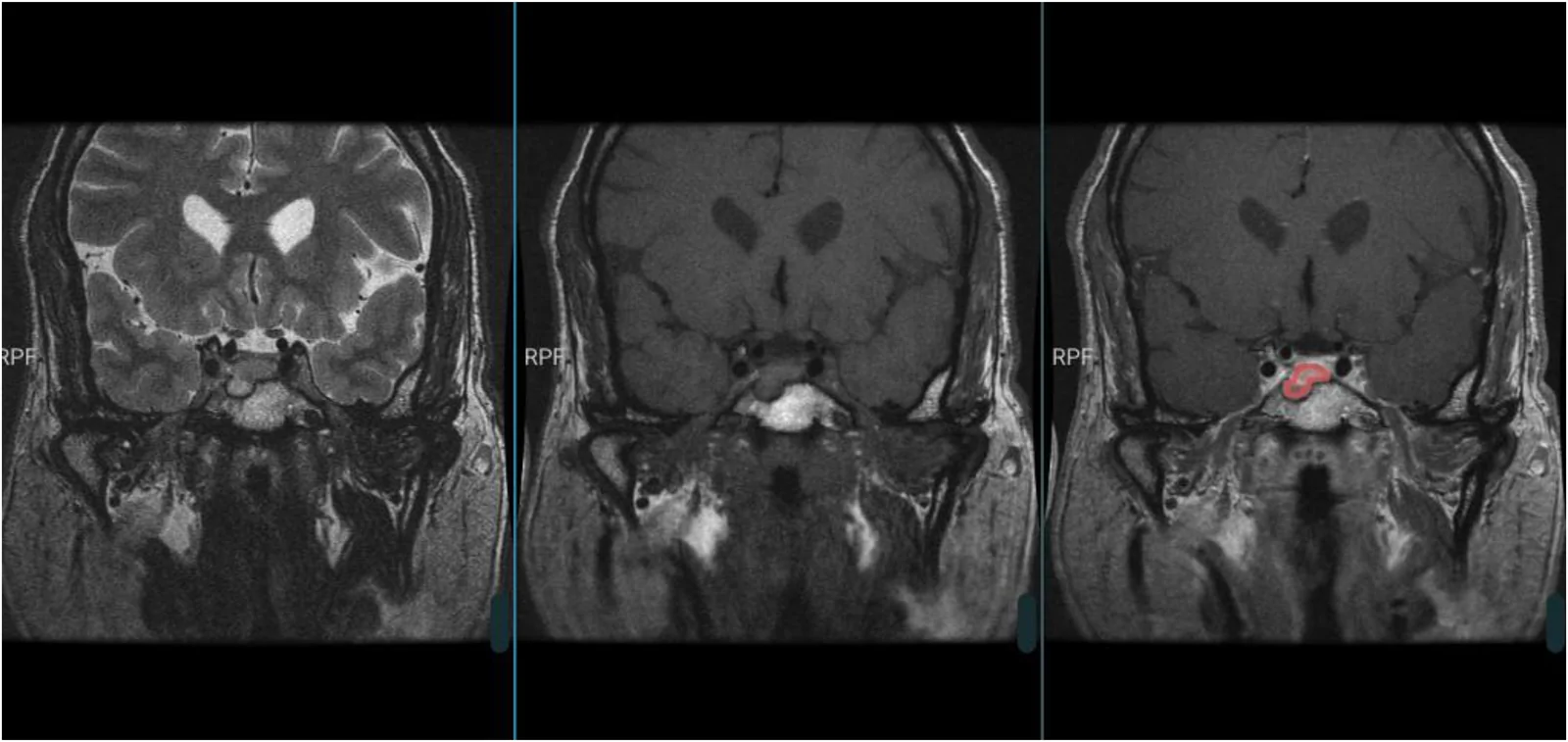
Magnetic resonance imaging of patient 3 showing a Knosp 2 invasive pituitary adenoma to the right cavernous sinus with an isointense-hyperintense signal in the T2-weighted sequence. The tumor is also contacting the chiasm.

Pituitary MRI revealed a 2-cm maximal diameter macroadenoma contacting the optic chiasm, with some right-side cavernous sinus invasion classified as Knosp 2 class with isointensity-hyperintensity signal in T2 sequences (Fig. 5). Given the proximity to the optic apparatus, the tumor was considered potentially threatening to vision. Optic coherence tomography and campimetry examination indicated no chiasmatic functional damage. The T2-weighted imaging showing isointense-hyperintense signal suggested a sparsely granulated phenotype and potential resistance to fgSRLs.

#### Functional testing: short acute octreotide test

The sAOT showed partial GH suppression: baseline GH of 12.1 ng/mL decreased to 2.5 ng/mL at 2 hours. This result falls into an intermediate range, not clearly predictive of either full fgSRL responsiveness or complete resistance, and in these cases, a partial response to fgSRLs is frequently shown (16).

#### Therapeutic dilemma and decision-making

This patient presented a complex therapeutic challenge. On the one hand, tumor proximity to the optic chiasm favored early surgical intervention. On the other hand, severe clinical symptoms, poor metabolic control, and high hormonal burden argued for rapid biochemical normalization before surgery. Moreover, imaging and functional biomarkers suggested that fgSRL monotherapy might be insufficient to achieve timely control as this case also presented hyperprolactinemia in a range that was very suggestive of a mixed GH-PRL tumor. As recently confirmed in a large Spanish series, somatoprolactinoma cases have demonstrated an even poorer response to fgSRL monotherapy of about 30% (36, 37).

#### Rationale for preoperative medical therapy

Preoperative medical therapy in acromegaly has historically been controversial. While some studies failed to show clear surgical benefit, others demonstrated improved anesthetic safety, reduced soft tissue swelling, and, in selected cases, tumor shrinkage. In addition, preoperative treatment may represent a useful therapeutic tool when surgery is delayed for any reason, allowing biochemical control and clinical stabilization while definitive surgical management is deferred (38, 39). Within a precision-medicine framework, preoperative therapy can be particularly valuable in patients with high tumor burden and severe systemic manifestations, provided that the biomarkers used are accurate enough. Pasireotide monotherapy was considered due to its high effectiveness in inducing tumor shrinkage and the isointense-hyperintense T2 signal (14, 15, 40) along with the potential insufficient responsiveness to fgSRLs in this case (41, 42). However, after discussing the options with the patient, the poor glycemic control situation played against the use of this compound as a first-line option.

#### Precision-guided combination therapy

In this case, preoperative combination therapy was decided using octreotide LAR 20 mg monthly and pegvisomant at 1.5 mg/kg/week, administered every other day. Cabergoline at 2 mg/week was added to complement the treatment, given the very high possibility that the tumor was a mixed GH-PRL adenoma. The rationale for this combination therapy was 3-fold: a) rapid biochemical control through GH receptor blockade with pegvisomant; b) potential tumor-stabilizing or shrinking effect mediated by somatostatin receptor activation; and c) avoidance of prolonged ineffective fgSRL monotherapy in a patient with intermediate biomarkers, in whom the coexistence of hyperprolactinemia at concentrations higher than those merely explained by stalk compression but the existence of a mixed GH-PRL tumor precluded even a poor response to fgSRL monotherapy.

Within 8 weeks, the patient’s IGF-1 normalized, GH levels decreased substantially, testosterone levels recovered to normal values, and he reported dramatic improvement in headaches, sweating, sexual activity, and fatigue. Glycemic control improved sufficiently to allow reduction of insulin doses while dapagliflozin 10 mg/day was kept to enhance cardiac protection.

#### Imaging response and surgical outcome

A repeated MRI after 6 months of combination therapy demonstrated a 20% reduction in tumor volume, with decreased suprasellar extension and reduced contact with the optic chiasm. These changes facilitated further safer surgical resection. Transsphenoidal surgery was subsequently performed without complications. The pathologist report indicated a mixed GH-PRL immunopositive tumor with intermediate to low staining for E-cadherin and low SSTR2. Additional molecular studies were performed showing high expression of DR2 as well as low SSTR5.

Postoperatively, residual tumor was minimal, with a remnant of 0.4 cm apparently adhered to the right cavernous sinus, and adjuvant therapy was individualized based on biochemical results and tumor phenotype by using the same 3-drug combination at lower doses, particularly in the case of pegvisomant, which was given at 72-hour intervals. During the follow-up discussion with the patient on the potential of curative treatment, radiotherapy was discarded and as the remnant seemed surgically accessible, another transsphenoidal surgical procedure was performed and hormonal remission confirmed shortly after it.

#### Clinical implications

This case illustrates how intermediate phenotypes—characterized by heterogeneous imaging and functional biomarkers—benefit from flexible, individualized strategies rather than rigid algorithms. The ACROFAST framework explicitly accommodates such scenarios, allowing medical combination therapy to be deployed early when intense clinical symptoms and tumor burden justify a timely proactive approach.

## Discussion: precision medicine–driven selection and sequencing of medical therapy in acromegaly, including the emerging role of pasireotide

### From empirical algorithms to biologically informed decision-making

The management of acromegaly has historically relied on relatively standardized, stepwise algorithms in which transsphenoidal surgery is followed, when needed, by fgSRLs, with subsequent escalation or switching based on biochemical response assessed over time (3). While this approach has undoubtedly improved outcomes compared with earlier eras, it implicitly assumes biological homogeneity among somatotroph PitNETs and often results in prolonged periods of uncontrolled disease in patients with intrinsically resistant tumors.

This limitation is increasingly recognized as clinically relevant. Persistent exposure to excess GH and IGF-1 contributes to irreversible cardiovascular remodeling, progressive metabolic dysfunction, osteoarticular disease, and impaired QoL (2, 5). Consequently, there has been a paradigm shift toward earlier identification of the most effective therapy for a given patient, rather than sequential empirical trials.

Precision medicine in acromegaly aims to address this challenge by integrating tumor biology, functional responsiveness to available compounds, and clinical context into therapeutic decision-making, by using biomarkers with strong predictive value; Table 1 summarizes the most relevant biomarkers described for identification of response to fgSRLs described and validated to date (43). The ACROFAST study provides prospective evidence that such an approach is feasible and improves the efficiency of disease control. Different initiatives have recently highlighted the necessity of establishing prognostic classification of somatotropic and other PitNETs, allowing a more efficient medical treatment algorithm (44, 45). Our approach, in which roughly 3 subclasses of acromegaly patients could be defined, are in agreement with previous cluster analyses (46). Our ACROFAST algorithm was designed to overcome the lack of control in more than 50% of cases usually observed when fgSRLs are given irrespective of the patient's characteristics. In the ACROFAST study nearly an 80% hormonal control was achieved when a personalized protocol was used. However, as acromegaly therapeutics continue to evolve, it is essential to consider how agents not included in ACROFAST—most notably pasireotide—may be integrated into this framework; thus, a next study including pasireotide is warranted.

**Table 1:** Main predictive markers used in precision medicine-guided treatment selection in acromegaly

Marker	Timing of use	Main predictive value	Favorable pattern	Unfavorable pattern	Main limitations	Main therapeutic implication
T2-weighted MRI signal intensity	Preoperative	Predicts likelihood of response to fgSRLs; may also help identify candidates for pasireotide in selected settings	T2-hypointense	T2-isointense/hyperintense	Qualitative assessment may vary; not fully deterministic	Supports fgSRL-first approach in favorable cases; supports earlier alternative strategies in unfavorable cases
sAOT	Preoperative and selected postoperative contexts	Functional estimate of acute GH suppressibility and likely response to fgSRLs	Strong GH suppression	Poor GH suppression	Interpretation depends partly on baseline GH; postoperative meaning may differ; fixed cutoffs should not be used rigidly	Helps stratify likely full responders vs poor responders to fgSRLs
E-cadherin expression	Postoperative	Correlates with tumor differentiation and sensitivity to fgSRLs	Preserved/high expression	Low expression	Requires tissue; not available preoperatively	Supports postoperative refinement of medical selection
SSTR2 expression	Postoperative	Predicts likelihood of response to fgSRLs	High expression	Low expression	Requires tissue; assay variability	Supports fgSRL use when preserved
SSTR5 expression	Postoperative	May support consideration of pasireotide in selected cases	Higher expression	Low expression	Not yet fully validated prospectively	May favor pasireotide in selected resistant cases
AIP status/AIP expression	Postoperative (and genetic context)	Associated with aggressive phenotype and lower fgSRL responsiveness in many cases	Wild-type/preserved expression	Pathogenic mutation/low expression	Not universal; variable phenotype	Supports earlier non-fgSRL strategies in appropriate cases
Granulation pattern	Postoperative	Broad biological predictor of treatment responsiveness	Densely granulated	Sparsely granulated	Requires pathology	

Abbreviations: fgSRLs, first-generation somatostatin receptor ligands; GH, growth hormone; MRI, magnetic resonance imaging; sAOT, short acute octreotide test; SSTR2, somatostatin receptor subtype 2.

### First-generation somatostatin receptor ligands: defining the true responders

FgSRLs remain the cornerstone of medical therapy for acromegaly and are highly effective in a biologically defined subset of patients. Newer SSTR2-directed compounds, including CAM2029 and paltusotine, may in the future be incorporated into the same biomarker-guided therapeutic branch as fgSRLs, although this extrapolation still requires prospective validation. Because their principal mechanism remains focused on SSTR2 signaling, the same biological framework may be applicable, but treatment-specific validation is still needed. Their antisecretory and antiproliferative effects are mediated primarily through SSTR2. Numerous studies have demonstrated that patients with densely granulated tumors, high SSTR2, E-cadherin, and AIP expression, as well as favorable imaging and functional biomarkers, have a high likelihood of achieving biochemical control with fgSRL monotherapy and these biomarkers are currently easily implementable in pituitary centers of excellence.

The integration of T2-weighted MRI signal intensity and the sAOT allow clinicians to identify the degree of fgSRLs responsiveness at diagnosis. As illustrated by the case of patient 2, concordantly favorable biomarkers justify confident use of fgSRL monotherapy, even in scenarios where surgery is delayed or declined. In such patients, fgSRLs remain an optimal and well-tolerated long-term strategy.

### Pegvisomant: prioritizing rapid and reliable biochemical control

Pegvisomant, a GH receptor antagonist, offers the most reliable and effective biochemical control in acromegaly, independent of tumor receptor expression profile or granulation pattern. Large observational studies and registries have demonstrated normalization of IGF-1 in the majority of treated patients when doses are appropriately titrated (47), but it does not address tumor burden.

Within a precision-medicine framework, pegvisomant is particularly valuable for patients with predicted resistance to fgSRLs, including those with T2-hyperintense tumors, poor GH suppression in the sAOT, and postsurgical immunohistological information indicating low SSTR2 expression and reduced E-cadherin staining. In such cases, early initiation of pegvisomant avoids prolonged ineffective therapy and minimizes cumulative exposure to excess GH and IGF-1, as illustrated by the case of patient 1.

The concerns regarding tumor growth during pegvisomant therapy have been largely mitigated by long-term data showing that clinically relevant progression is very uncommon and typically reflects underlying tumor biology rather than a direct drug effect, provided that regular MRI monitoring is performed (47).

### Combination therapy: addressing both hormonal excess and tumor burden

Combination therapy with an fgSRL and pegvisomant represents a rational strategy in patients with high tumor burden, intermediate biomarker profiles, or partial responsiveness to fgSRLs. This approach relies on complementary mechanisms of action: FgSRLs may contribute to tumor stabilization or shrinkage, while pegvisomant ensures robust biochemical control. This combination has a proven high efficacy, and theoretically, would be the most effective option for all patients without any other consideration of subclassification, although there would still be 50% of full responders to fgSRLs not requiring the addition of pegvisomant to reach hormonal control, and the cost-effectiveness evaluation of such a treatment modality would be a constraint. Thus, the identification of potential full or partial responders to fgSRLS is important to achieve the best possible personalized approach, which also takes into account cost-effectiveness (48, 49).

In the ACROFAST study, combination therapy was incorporated as an early option for patients with discordant or intermediate biomarkers, demonstrating rapid IGF-1 normalization and favorable clinical outcomes. Patient 3 exemplifies this scenario, in which preoperative combination therapy achieved both biochemical control and significant tumor volume reduction, facilitating safer surgical intervention.

Finally, regarding combination therapy, the addition of cabergoline on top of fgSRLs has some clinical utility in contemporary management. Although its biochemical efficacy is generally quite more modest than that of fgSRLs, pegvisomant, or pasireotide, it is inexpensive, widely available, and may be useful more frequently rather than as monotherapy, as adjunctive therapy not only in mixed GH-PRL tumors but also in selected patients with mild residual biochemical activity. However, in those countries where neither pasireotide nor pegvisomant are available, the possibility of performing true precision medicine is quite limited as many patients will fall into a noncontrolled hormonal condition; cabergoline maybe helpful in these countries in the aforementioned cases where a mixed GH-PRL is present, and as adjunctive to fgSRLs in patients with very modestly elevated IGF-1.

### Pasireotide: defining its role within a precision medicine framework

Pasireotide, the second-generation somatostatin multireceptor ligand with a high affinity for SSTR5—as well as for SSTR3—and a lower relative affinity for SSTR2, represents an important addition to the therapeutic armamentarium in acromegaly. In randomized clinical trials, pasireotide demonstrated superior biochemical efficacy in selected populations, particularly in patients previously uncontrolled on fgSRLs, achieving an effectiveness in more than 60% of patients in real-life experience (41, 42).

However, pasireotide was not included in the ACROFAST trial, as no predictive biomarkers of pasireotide treatment were available at the time of the study design and their definitions are still awaited. However, since then, new evidence indicates that T2-hyperintense signal on MRI could be a predictor of good response to pasireotide (14, 15, 50) and thus, it could be included in a general algorithm of personalized medicine in acromegaly patients, particularly when tumor burden is of importance (51), although validation of these imaging biomarkers and those of a molecular nature are still required.

If the sAOT may strongly identify response capacity to fgSRLs, pasireotide is still lacking a functional test as the one for fgSRLs. The development of such a procedure in which subcutaneous pasireotide will be used with determination of GH at 2 hours is therefore required to identify the 60% of patients who will respond to pasireotide, but also the 40% not responding to the compound; data from early studies with the subcutaneous compound (52) indicated a suppressive effect of GH at 2 hours after its administration. Thus, the definition of a 2- hour GH cutoff will be very valuable for predicting a full long-acting pasireotide response.

**Figure 6:**
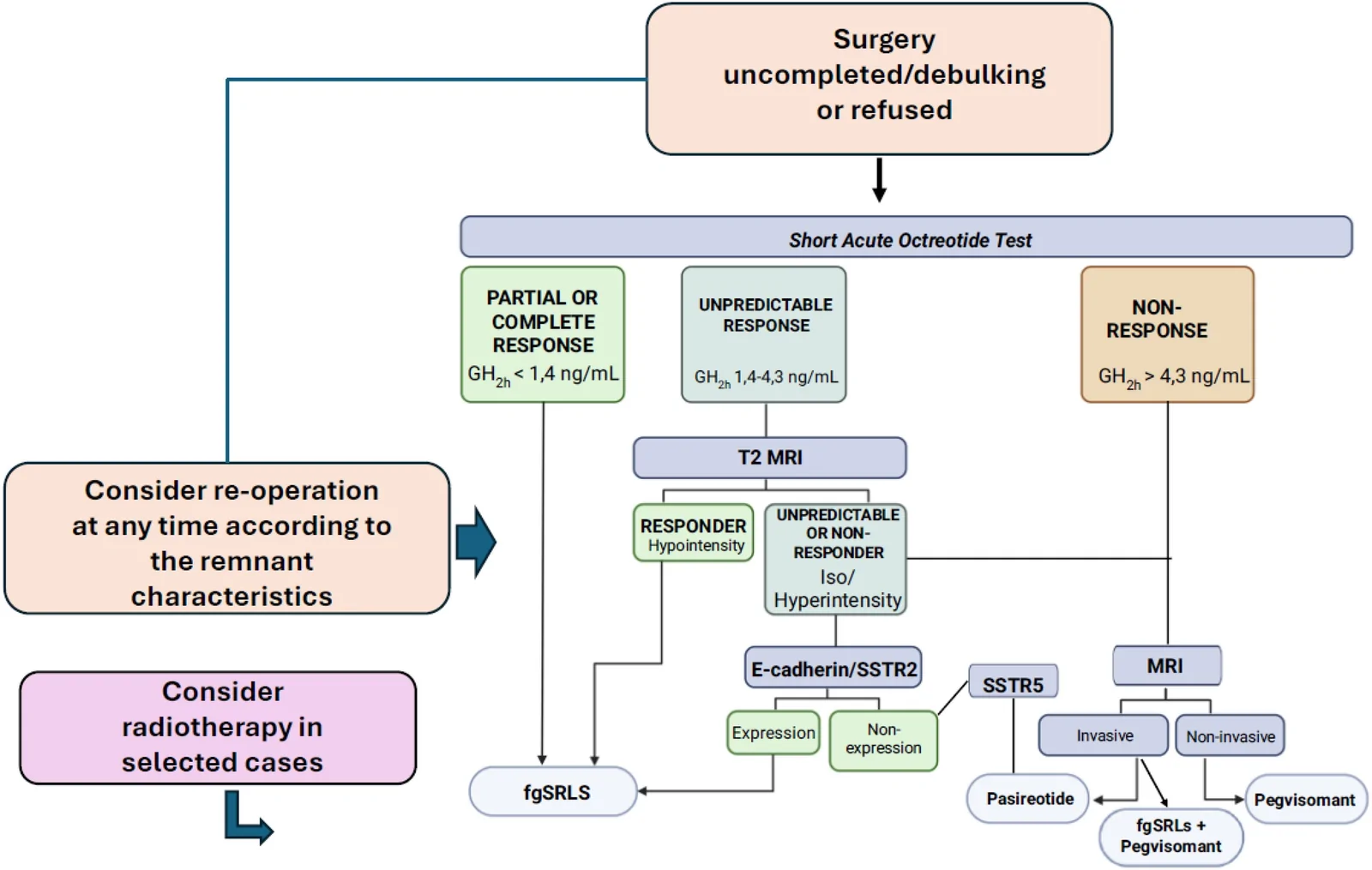
Proposed algorithm for decision of medical treatment modality. Surgery is the first option either with curative intention or for debulking purpose. Radiotherapy could be offered as final curative option. Medical treatment is offered when surgery is noncurative, or in certain cases as preoperative therapy, or as primary treatment in those cases not suitable for surgery. The algorithm for deciding the use of first-generation somatostatin receptor ligands as the first option or other compounds is based on the short acute octreotide test results and the evaluation of magnetic resonance imaging T2 signal intensity obtained by qualitative assessment and when tumor tissue is available on the evaluation of E-cadherin, SSTR2, and SSTR5 expression.

Therefore, today, precision medicine with response predictors must be restricted to fgSRL compounds as shown in the algorithm presented in Fig. 6 that could be implemented at the time of diagnosis or after surgical failure complementing the functional and imaging information with molecular profiling.

Emerging evidence suggests that pasireotide responsiveness may be associated with tumor characteristics distinct from those predicting fgSRL response. Tumors with low SSTR2 expression, sparse granulation, and potentially high SSTR5 expression may derive greater benefit from pasireotide, but these biomarkers are still to be challenged in prospective trials. When surgical treatment is insufficient and even considering those cases in which a debulking surgery is the goal, the opportunity to examine the molecular profile is currently important. For fgSRLs a combination of high expression of E-cadherin, SSTR2, and *AIP* confers the highest probability of a complete response and could also identify partial responders for whom combination therapy or eventually, according to the tumor burden, pasireotide would be recommended; this latter is currently indicated as per clinical judgment (51).

Despite the valuable good results of pasireotide in fgSRL-resistant cases, its clinical use is still constrained by its metabolic adverse effect profile, particularly the high incidence of hyperglycemia and worsening of diabetes reported in about 50% of treated patients as also exemplified in patient 3, in whom this issue was taken into consideration when discussing therapeutic options. However, managed by experienced endocrinologists, these metabolic complications do not necessarily represent a major clinical limitation (41, 42). In the eventuality that a patient develops a very unstable hyperglycemic situation, a careful evaluation of the risks and benefits is required for not giving pasireotide to some patients with acromegaly, another condition which is associated with a reduction in life expectancy and increase in standardized mortality ratio.

Consequently, pasireotide may be best suited for patients with predicted resistance to fgSRLs, and particularly for those with significant tumor burden where tumor-directed effects are desirable; the most favorable clinical profile may include an acceptable baseline metabolic risk and in those cases with previous diabetes, the patient will need to have sufficient skills to manage intensive insulin treatment. In such patients, pasireotide may represent an alternative to combination therapy with fgSRLs and pegvisomant, particularly when tumor shrinkage is prioritized. A recurring challenge in acromegaly management is balancing the goals of biochemical control and tumor size reduction. Precision medicine reframes this issue by aligning therapeutic priorities with individual patient and tumor characteristics rather than imposing a single hierarchy of outcomes. In patients with invasive or nonresectable tumors, agents with antiproliferative potential such as fgSRLs and particularly pasireotide have to be prioritized. In contrast, in patients in whom biochemical control is the dominant concern and tumor growth risk is low, pegvisomant may be favored in the treatment modality. Combination therapy provides a flexible means of addressing both objectives simultaneously in selected cases. However, head-to-head comparative data between these strategies on a precision medicine setting are still lacking.

### Integrating the patient into contemporary precision algorithms

While ACROFAST provides a validated framework for biomarker-guided therapy using fgSRLs, the new SSTR2 targeted compounds and pegvisomant, future iterations of precision algorithms may incorporate pasireotide as additional predictive tools become available. But above all, modern medicine, and in particular precision medicine based on biomarkers of therapeutic response, must also include the discussion of therapeutic options and their outcomes prediction with the patient, who should be involved in the therapeutic decision-making process. In this sense, precision medicine in acromegaly should not only be personalized and predictive, but should also include a fourth “P,” that is, the P of participatory. As clearly indicated in this article, in all the cases presented in this paper, despite using biomarkers to help in guiding the best possible treatment for each specific case, the final decision was shared with each patient. Finally, in real-world practice, biomarker-based prediction must be integrated also with nonbiological determinants of treatment feasibility and effectiveness, including adherence, injection burden, home or caregiver support, patient acceptance, relevant comorbidities, hepatic and renal function, drug-drug interactions, and treatment access or cost. Therefore, the biologically “best” option may not always be the most appropriate practical option for a given patient or just may not be accepted by the patient. In this sense, precision medicine should be understood as the integration of biological predictors with individualized clinical and therapeutic characteristics but also including the specific social and personal context of the patient.

### Future prospects

An additional future direction for precision medicine in acromegaly is also the integration of machine learning and artificial intelligence (AI) tools capable of combining clinical, biochemical, radiological, pathological, and molecular data into composite predictive models. This discipline of scientific knowledge is clearly gaining ground in medical science and is moving toward providing tools that will undoubtedly contribute to better patient classification, particularly in the field of endocrinology and, of course, also in acromegaly, a paradigm of disease of a heterogeneous nature. This will allow for better classification of subgroups based on clinical-biological behavior and prediction of therapeutic response. In recent years, several groups, including our own, have presented studies that clearly support the value that can be obtained from applying AI tools to the management of acromegaly (53, 54). However, these approaches should currently be considered complementary rather than definitive, as they still require external validation, prospective assessment, and adequate interpretability before routine implementation in daily practice. Finally, radiomics is emerging as a promising tool to extract quantitative imaging features that are not visible to the human eye, enabling a more objective characterization of pituitary tumors. In acromegaly, radiomics-based analysis of MRI has the potential to improve the prediction of tumor biological behavior and therapeutic response, particularly to SRLs, thereby contributing to more personalized treatment strategies (55). The integration of radiomics with clinical and molecular data may become a key component of precision medicine approaches in pituitary neuroendocrine tumors (56, 57). Thus, AI-supported tools for classification/scoring systems aiming to define risk stratification, although not replacing individualized clinical judgment and patient-shared decisions, will be very helpful in the decision-making process in the near future.

## Conclusion

Precision medicine has become an actionable reality in acromegaly. By integrating imaging, functional testing, and tumor phenotype, clinicians can individualize therapy, optimize sequencing, and reduce time to effective disease control. The ACROFAST strategy provides a robust foundation for this approach. Pasireotide, although not included in ACROFAST, represents an important complementary option that may be incorporated into precision algorithms for selected patients, provided that validation trials such as the ACROFAST II study finally confirm its best indication for selected patients. As predictive tools evolve, the rational integration of all available therapies will further refine personalized care in acromegaly.

## Data Availability

Data sharing is not applicable to this article as no datasets were generated or analyzed for this paper. Restrictions apply to the availability of some data analyzed during the generation of this paper to preserve patient confidentiality.

## Abbreviations

ACROFAST study: Acromegaly Clinical Research on Outcomes with Functional and Stratified Therapy
AI: artificial intelligence
DPP-4: dipeptidyl peptidase 4
fgSRLs: first-generation somatostatin receptor ligands
GH: growth hormone
HbA_1c_: glycated hemoglobin A_1c_
IGF-1: insulin-like growth factor 1
MRI: magnetic resonance imaging
PitNET: pituitary neuroendocrine tumor
PRL: prolactin
QoL: quality of life
sAOT: short acute octreotide test
SSTR2: somatostatin receptor subtype 2
ULN: upper limit of normal
